# Abnormalities of Electroencephalography Microstates in Drug-Naïve, First-Episode Schizophrenia

**DOI:** 10.3389/fpsyt.2022.853602

**Published:** 2022-03-14

**Authors:** Qiaoling Sun, Linlin Zhao, Liwen Tan

**Affiliations:** Department of Psychiatry, National Clinical Research Center for Mental Disorders, China National Technology Institute on Mental Disorders, The Second Xiangya Hospital of Central South University, Changsha, China

**Keywords:** drug-naïve, first-episode schizophrenia, resting-state, EEG, microstate

## Abstract

**Objective:**

Microstate analysis is a powerful tool to probe the brain functions, and changes in microstates under electroencephalography (EEG) have been repeatedly reported in patients with schizophrenia. This study aimed to investigate the dynamics of EEG microstates in drug-naïve, first-episode schizophrenia (FE-SCH) and to test the relationship between EEG microstates and clinical symptoms.

**Methods:**

Resting-state EEG were recorded for 23 patients with FE-SCH and 23 healthy controls using a 64-channel cap. Three parameters, i.e., contribution, duration, and occurrence, of the four microstate classes were calculated. Group differences in EEG microstates and their clinical symptoms [assessed using the Positive and Negative Syndrome Scale (PANSS)] were analyzed.

**Results:**

Compared with healthy controls, patients with FE-SCH showed increased duration, occurrence and contribution of microstate class C and decreased contribution and occurrence of microstate class D. In addition, the score of positive symptoms in PANSS was negatively correlated with the occurrence of microstate D.

**Conclusion:**

Our findings showed abnormal patterns of EEG microstates in drug-naïve, first-episode schizophrenia, which might help distinguish individuals with schizophrenia in the early stage and develop early intervention strategies.

## Introduction

Schizophrenia is a severe mental illness featured with complex etiology, long course, severe functional impairment, and poor prognosis; it usually occurs in early adulthood with a lifetime prevalence of approximately 1% (1, 2). This disease has been considered as one of the leading causes of disability worldwide (2, 3) and has brought a great burden to the patients, their families, as well as the health care systems (4–6). Evidence has suggested that prolonged periods of untreated psychosis can lead to adverse functional outcomes (7, 8). Therefore, a further understanding of schizophrenia in the early stage could provide information for early detection and intervention, which is crucial in reducing the risk for deterioration associated with the chronic and recurring process of the disease.

The pathophysiological hypothesis of schizophrenia has suggested that the occurrence of the disease might be due to poor connections across and within brain networks (9). Electroencephalography (EEG) is a method based on an integrative, complex, and *in vivo* model of brain functions, which can be used to analyze the neural synchrony associated with the pathophysiology of schizophrenia (10–12). The resting-state EEG has a high temporal resolution so that it can capture the rapid-changing dynamics of neural networks. The multichannel resting-state EEG signal can be parsed into a limited number of distinct quasi-stable states (13), which are defined by topographies of electric potentials recorded in a multichannel array over the scalp. A certain topographic map will remain stable for 80–120 ms before rapidly transitioning to another one. The quasi-stable period of this single topographic map is called EEG microstate. Four major microstate classes (i.e., class A–D) have been identified to explain a total of 65–84% of the variance of EEG data (14); they have shown high reliability and consistency across studies (15). EEG microstates were considered to be the most fundamental pattern of human neuronal functions (16) and were regarded as cornerstones of mental states in EEG data (17).

The abnormality of EEG microstates in patients with schizophrenia has been observed and reported in multiple previous studies (18–22), most of which involved patients with chronic conditions (19, 23, 24) or a mixed sample with no restriction on the course of disease (25–28). For schizophrenia, the early stage represents the transition from the premorbid to the morbid state (7); a clear diagnosis and early intervention during this period can help avoid the interference of long course and numerous attacks of the disease. Therefore, the involvement of first-episode patients with psychosis becomes necessary in studies on early-stage characteristics of schizophrenia. One study (29) found that patients with first-episode psychosis showed similar microstate dynamics as patients with chronic schizophrenia; both samples showed increased microstate C, decreased microstate D, and shortened duration of microstate B, as compared with healthy controls (HCs). However, another study (30) found an increased duration of microstate B in patients with first-episode psychosis. One possible explanation for this disparity might be the difference in medication between these studies.

Evidence suggested that some antipsychotic drugs can affect EEG microstates in both healthy individuals and patients with psychosis. The increase of microstate D has been reported in healthy individuals after the use of antipsychotic drugs (31); and in those with first-episode psychosis, some antipsychotic medications have a regulatory effect on microstate A and B, reducing the contribution of microstate A and increasing the contribution of microstate B (30). It has also been reported that antipsychotics could regulate the abnormal microstate dynamics with good efficacy (29). For instance, patients with schizophrenia showed decreased occurrence and increased duration of microstate C after the use of antipsychotic medications (22). Furthermore, antipsychotic drugs can increase the duration of microstates in a non-specific manner (31), e.g., sulpiride (32) and haloperidol (31) can increase the average duration of all microstate classes. Therefore, medication should be taken into account in studies on EEG microstates.

Koenig et al. (33) recruited drug-naïve, first-episode schizophrenia (FE-SCH) for the first time to eliminate the impact of medication. This study found that compared with HCs, the microstate class D was decreased and the occurrence of microstate A was increased. Lehmann et al. (34) subsequently repeated their study and, in addition to confirming the former results, found a decrease in the duration of microstate B and an increase in the incidence of microstate C. As the results of previous studies have been inconsistent so far. The primary aim of the current study was to replicate these finding using an independent dataset to investigate the characteristics of EEG microstates in FE-SCH and to explore the relationship between the EEG microstates and psychopathological symptoms of the patients.

## Materials and Methods

### Participants

Twenty-three patients with FE-SCH from the Second Xiangya Hospital of Central South University and 23 gender- and age-matched HCs were included. All the patients met the criteria for schizophrenia in the International Classification of Diseases (10th Edition) and were screened with the use of Mini-International Neuropsychiatric Interview. Inclusion criteria for patients were: (1) aged between 18 and 60 years, (2) drug-naïve, and (3) right-handed. The HCs were recruited from the community, with the inclusion criteria of the absence of a current or lifetime diagnosis of Axis I or II disorders. Exclusion criteria for both groups were: (1) severe physical disorders, neurological disorders, substance abuse or dependence, and intellectual disability; (2) being drunk or having taken benzodiazepines in the last 24 h; and (3) unable to provide informed consent.

All the participants were fully informed of the procedures and signed the informed consent form. The study was approved by the Clinical Research Ethics Committee of the Second Xiangya Hospital, Central South University. Information of socio-demographic variables such as age, education and gender were collected from all individuals. Positive and Negative Syndrome Scale (PANSS) (35), which comprises of 30 items in three subscales (positive symptoms, negative symptoms, and general psychopathological symptoms), was applied to assess the severity of symptoms in the patients.

## Electroencephalography Recordings

The EEG activity was acquired using a 64 Brain Amp cap (Brain Products GmbH, Munich, Germany), with electrodes positioned according to the 10–20 International System and reference to linked mastoids. The vertical electrooculogram (VEOG) were recorded from the electrode beneath the right eye. The sampling frequency of the signal is 1,000 Hz and all impedances of the electrodes were kept below 10 kΩ. The data were recorded in an electrically shielded, sound-attenuated and lit room. The participants were asked to sit quietly and accommodated in the environment for about 3 min. The resting-state EEG data were collected with their eyes closed for 3–7 min.

### Data Pre-processing

**FIGURE 1:**
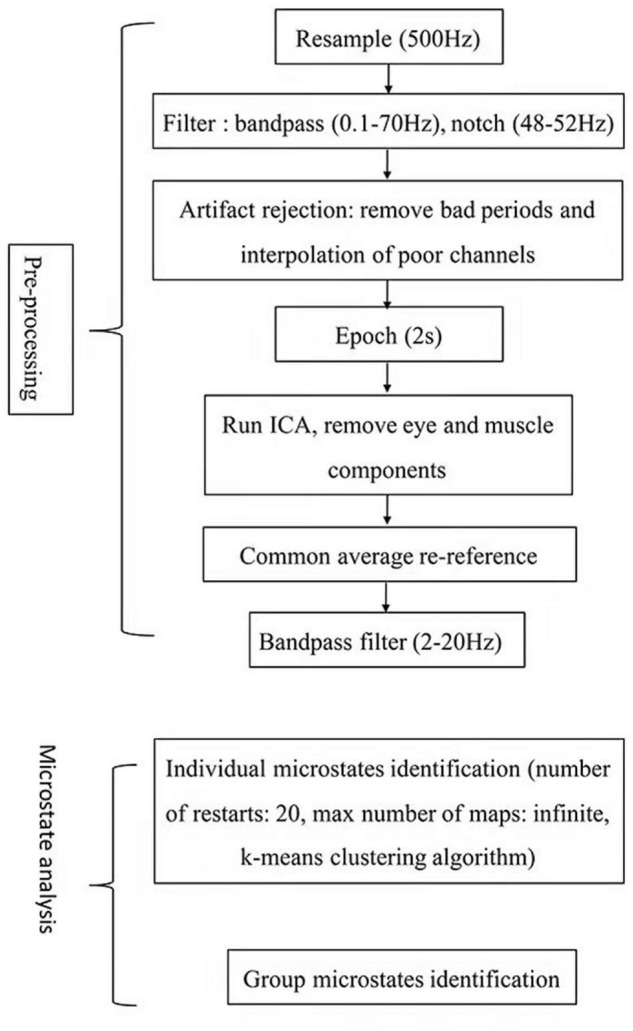
Flowchart detailing the pre-processing and data analysis steps.

Offline data were processed using the MATLAB-based open-source software package EEGLAB. The flowchart in Figure 1 illustrates the pre-processing and data analysis steps adopted in detail. First, resample the data to 500 Hz, the data were filtered with a bandpass of 0.1–70 Hz and a notch (48–52 Hz), and bad EEG periods were removed and channels with very poor signals were interpolated using spline interpolation. The data were then divided into 2 s segments, and eye and muscle movement artifacts were removed through independent component analysis. Finally, the data were re-referenced to the common average reference and bandpass filtered (2–20 Hz).

### Microstate Analysis

**FIGURE 2:**
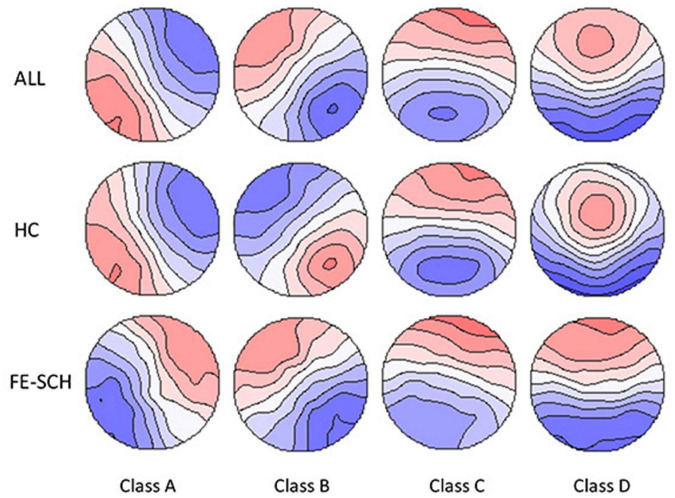
The spatial configuration of the four microstate classes for the two groups and all the participants. FE-SCH, drug-naïve, first-episode schizophrenia; HC, healthy control; ALL, all the participants in this study.

Microstate analysis was performed using the Microstate Analysis plugin developed by Thomas Koenig^1^. Individual microstate maps for each participant were calculated with four clusters preset. The number of repetitions was set at 20 and the max number of maps to include was set to infinite. In this study, a *k*-means clustering algorithm was used for microstate analysis. The group-level microstate classes were then identified for patients with FE-SCH and HCs separately. Using the mean microstate classes across all the participants as the template (Figure 2), maps at the individual and group levels were fitted, and the following parameters based on the mean microstate maps across subjects were extracted for the four microstate classes: contribution (the proportion of time for each microstate), duration (the average duration of a microstate class in milliseconds), and occurrence (total number of the microstates of a given class per second).

### Statistical Analysis

Statistics were computed using SPSS Version 23.0. Continuous variables were compared between the two groups using *t*-test, and sex distribution was compared using Pearson’s χ^2^ test. Repeated measures analysis of variance (rm-ANOVA) was applied to analyze inter-group differences, with microstate parameters (contribution, occurrence, and duration) and microstate classes (Class A, B, C, and D) as within-subject factors and group (FE-SCH and HCs) as between-subjects factors. Greenhouse–Geisser correction was applied for multiple comparisons. If significant group main effects or interactions were found in the rm-ANOVA, univariate ANOVA was then performed to investigate the simple effects.

We also investigated the relationships between microstate parameters and the score of PANSS (positive symptoms, negative symptoms, general psychopathological symptoms, and the total score) in the patient group using the Pearson’s correlation analysis.

## Results

### Subject Characteristics

The demographic, psychometric, and clinical characteristics of HCs and patients with FE-SCH were summarized in Table 1. There was no significant inter-group difference in sex, age, and level of education.

**TABLE 1:** Demographic and clinical characteristics of all participants (Mean ± SD).

	FE-SCH (*n* = 23)	HC (*n* = 23)	χ^2^/*t*	*p*
Sex (male/female)	16/7	13/10	0.840	0.359
Age (years)	28.04 ± 5.42	26.39 ± 6.95	0.899	0.373
Years of education	13.36 ± 2.40	14.14 ± 1.91	−1.181	0.244
Age at onset (years)	27.48 ± 5.54	–		
Illness duration (months)	8.14 ± 5.78	–		
PANSS total score	64.09 ± 16.80	–		
PANSS positive symptoms	16.96 ± 5.71	–		
PANSS negative symptoms	14.43 ± 7.67	–		
PANSS general psychopathological symptoms	32.08 ± 16.80	–		

*SD, standard deviation; PANSS, Positive and Negative Syndrome Scale; FE-SCH, first-episode, drug-naïve schizophrenia; HC, healthy control.*

### Inter-Group Comparison of Electroencephalography Parameters

There was no difference in the number of 2-s epochs after the exclusion of artifacts (*t* = 0.042; *p* = 0.0967) between HCs and patients with FE-SCH (Mean ± SD, 109.43 ± 37.302 and 109.83 ± 24.194, respectively). The four microstate classes explained 75 and 79% of the EEG variance for HCs and patients with FE-SCH, respectively.

**FIGURE 3:**
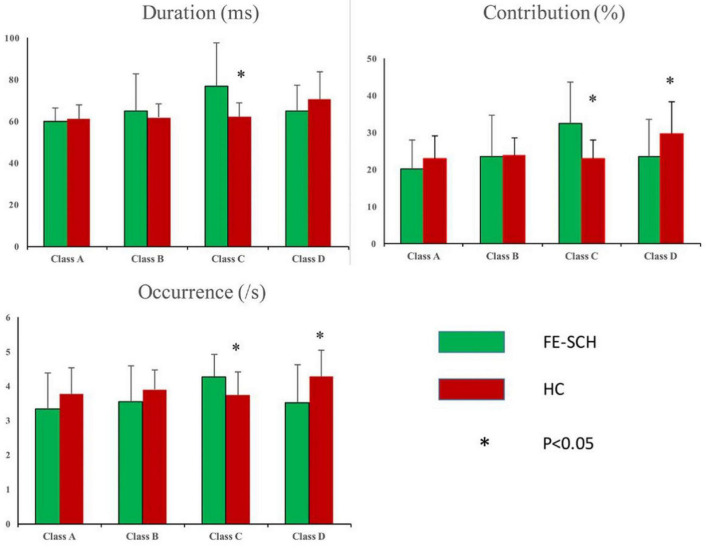
Inter-group comparison of microstate parameters: duration, contribution, and occurrence. FE-SCH, drug-naïve, first-episode schizophrenia; HC, healthy control.

Rm-ANOVA revealed an interaction of microstate parameter × microstate class × group (*F*_(2.859,125.813)_ = 5.071, *p* = 0.003), with differences in duration (*F* = 10.662, *p* = 0.002), contribution (*F* = 14.213, *p* < 0.001), and occurrence (*F* = 7.251, *p* = 0.010) of microstate class C, and contribution (*F* = 5.262, *p* = 0.027) and occurrence (*F* = 7.259, *p* = 0.010) of microstate class D (Figure 3). The means and standard deviations of the three parameters and four microstate classes of the two groups, together with the results of the *post hoc* univariate test, are presented in Table 2.

**TABLE 2:** *Post hoc* univariate test results for the three parameters of microstate classes (Mean ± SD).

Variable	Group		*F*	*p*	Partial η^2^
	FE-SCH	HC			
**Duration (ms)**					
A	60.12 ± 6.35	61.30 ± 6.48	0.388	0.537	0.009
B	64.81 ± 17.91	61.89 ± 6.41	0.541	0.466	0.012
C	77.07 ± 20.88	62.12 ± 6.79	10.662	0.002*	0.195
D	64.82 ± 12.68	70.51 ± 13.12	2.233	0.142	0.048
**Contribution (%)**					
A	20.35 ± 7.64	23.15 ± 6.04	1.902	0.175	0.041
B	23.53 ± 11.29	23.94 ± 4.72	0.026	0.872	0.001
C	32.58 ± 11.06	23.04 ± 5.00	14.213	<0.001***	0.244
D	23.54 ± 10.21	29.87 ± 8.41	5.262	0.027*	0.107
**Occurrence (/s)**					
A	3.34 ± 1.04	3.77 ± 0.77	2.502	0.121	0.054
B	3.54 ± 1.06	3.91 ± 0.57	2.149	0.150	0.047
C	4.27 ± 0.65	3.74 ± 0.67	7.251	0.010*	0.141
D	3.53 ± 1.09	4.28 ± 0.76	7.259	0.010*	0.142

*FE-SCH, first-episode, drug-naïve schizophrenia; HC, healthy control.*

*p < 0.05, ***p < 0.001.

### Relationship Between Microstate Parameters and Symptoms

**FIGURE 4:**
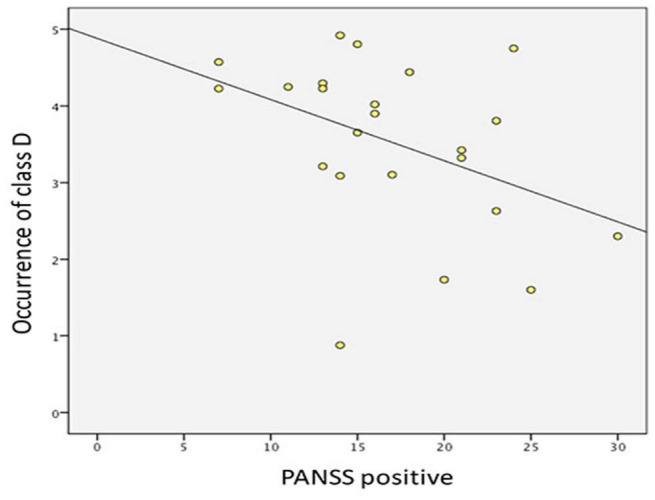
Correlation between PANSS positive symptoms and the occurrence of microstate class D in drug-naïve, first-episode patients with schizophrenia.

The result showed that the score of PANSS positive symptoms was negatively correlated with the occurrence of microstate class D (*r* = −0.416, *p* = 0.048) (Figure 4). No correlation was found between microstate parameters and other relevant scores of PANSS (i.e., the total score, the score of negative symptoms, and the score of general psychopathological symptoms).

## Discussion

It was an independent laboratory replication study. Through exploring the abnormalities of EEG microstates in FE-SCH, we found that, compared with HCs, the occurrence, duration, and contribution of microstate class C significantly increased, and the occurrence and contribution of microstate class D significantly decreased in patients with FE-SCH. For the relationship between clinical symptoms and EEG microstates, we found that the score of PANSS positive symptoms was negatively correlated with the occurrence of microstate class D.

Our study found increased microstate class C and decreased microstate class D in patients with FE-SCH, which is consistent with some previous studies (20, 29, 34). Such abnormalities of microstate class C and D have been identified in patients with chronic schizophrenia (19, 36), individuals with ultra-high risk for psychosis (37, 38), and even siblings of patients with schizophrenia (29). Two meta-analyses (28, 29) published in recent years showed similar results that the occurrence of microstate class C increased and the occurrence of microstate class D decreased in patients with schizophrenia. Furthermore, some researchers have proposed the dynamics of resting-state EEG microstates, especially for microstate classes C and D, as the potential endophenotype of schizophrenia (28, 29).

Studies combining functional Magnetic Resonance Imaging (fMRI) and EEG showed that the characteristics of microstates overlapped with those of resting-state networks identified using fMRI (39–41), indicating that microstates might be closely related to resting-state functional networks. It has been found that microstate class C is closely related to the default mode network (39), which consists of the medial prefrontal, parietal, and temporal cortices (42). Default mode network is the neural basis of ego (43); it was found to be activated during internally oriented mental processes and to play an important role in self-referential thoughts and episodic memory extraction (14, 44). The microstate class C might be generated at the bilateral medial temporal gyrus and the lateral parietal lobe (36), which were found to be associated with self-experience in fMRI studies (45, 46). It was also considered that microstate class C was associated with the activation of the default mode network (14, 39), the abnormal activation of which has been repeatedly demonstrated in schizophrenia (47, 48). Therefore, the increase of microstate class C might explain the abnormalities in self-focus and the self-experience in schizophrenia.

Microstate class D was found to be associated with the dorsal attention network (39). The source of microstate class D was located in the frontoparietal area (49), which was associated with the successful execution of attention-related tasks (33) and was extensively interconnected with both default mode and dorsal attention networks (50). Attention deficit has been regarded as one of the prominent symptoms of schizophrenia. Numerous studies revealed that patients with schizophrenia had obvious deficits in information processing under conditions of high processing load and distraction (33, 51, 52). In fact, patients with schizophrenia demonstrated frontal lobe dysfunction (53), as well as reduced interaction and functional integration between the prefrontal cortex and other cortical and subcortical brain structures (9), which might result in the imbalance of neural activities of different sites. Therefore, the decrease of microstate class D might suggest the impaired function of the frontoparietal and dorsal attention networks in schizophrenia.

Some studies on event-related potential found that microstates were associated with specific functions of information processing (54, 55). Microstate class C and D were found to be associated with higher and lower attention levels, respectively. Therefore, we may infer that increased microstate class C and decreased microstate class D in patients with FE-SCH in this study might indicate an abnormal state of attention distribution. These microstate abnormalities have been explained in a meta-analysis as an imbalance between processes involving saliency and processes that integrate contextual information (28). Moreover, Kikuchi et al. (22) found that successful antipsychotic treatment not only mitigated attention and executive control impairments in schizophrenia, but also regulated the patterns of microstate class C and D.

No difference was found in microstates class A and B between the two groups. Although some studies found abnormalities of these two microstate classes (33, 34), conclusion cannot be reached with the existing literature due to the heterogeneity of studies. Two meta-analyses (28, 29) showed consistent results that there was no significant difference in the parameters of microstate A and B between patients with schizophrenia and HCs. Therefore, these two microstate classes might be of limited significance in the identification of FE-SCH.

Regarding the relationship between clinical symptoms and EEG microstates, we found that the score of PANSS positive symptoms was negatively correlated with the occurrence of microstate class D. To date, there have been consistent findings about the relationship between microstate class D and positive psychotic symptoms (20, 21, 28). A study found that the duration of microstate class D was negatively correlated with the score of paranoid-hallucinatory syndromes (33). Compared with HCs, the duration of microstate class D was shorter in those with schizophrenia (20), and this effect was particularly significant in patients with acute experience of hallucinations (21). Apart from microstate class D, microstate classes C and A were also demonstrated to be associated with positive symptoms in several studies. A study involving individuals with high risk for schizophrenia found a positive association between microstate class C and positive psychotic symptoms (38), but this result has not been reproduced in studies of FE-SCH. As for microstate class A, a study found that it was positively associated with the score of PANSS positive symptoms in patients with first-episode psychosis (30), but in another study, a positive association was present with negative symptoms (26).

Microstate class D was found to be associated with the reduction of positive symptoms. A study found that the indication of its occurrence was similar to the adjustment of strategy when erroneous processing occurred (21, 56). The positive symptoms might be related to the attribution of internally generated speech error to external source, and decreased occurrence of microstate class D might be associated with decreased ability to correct such errors. In addition, decreased occurrence of microstate class D might also be related to decreased activation of dorsal attention network in patients, leading to focal attention impairments and the co-activation of functionally incompatible networks (21), thereby resulting in positive symptoms.

Some limitations need to be mentioned in this study. First, this is a cross-sectional study, which precluded us to assess the severity and progression of the disease. Second, our study still shares the common shortcoming of a small sample size with many previous studies. Due to possible sampling error and disease heterogeneity, a small sample might produce insignificance or even opposite effects, thereby leading to inaccurate conclusions. Therefore, the sample needs to be expanded in future works. Third, this study only included the four main microstate classes, which could not cover the overall variance; thus, more microstate classes need to be included in future works for a better explanation of the total variance. However, the analysis of the four major microstate classes allows us to compare our findings with previous evidence directly. The four microstate classes explained 79% of the overall variance, indicating that our method is appropriate.

## Conclusion

In summary, our findings suggested that drug-naïve, first-episode patients with schizophrenia might be associated with increased microstate class C and decreased microstate class D. We also found a negative correlation between microstate class D and positive symptoms. These results are in line with previous studies. Furthermore, the deviant microstate class C and D might reflect the underlying pathogenesis of schizophrenia, which could provide useful information in the identification of patients with schizophrenia and the development of intervention strategies.

## Data Availability Statement

The original contributions presented in the study are included in the article/supplementary material, further inquiries can be directed to the corresponding author.

## Ethics Statement

The studies involving human participants were reviewed and approved by the Second Xiangya Hospital. Written informed consent to participate in this study was provided by the participants’ legal guardian/next of kin.

## Author Contributions

QS collected and analyzed the data. QS and LZ wrote the original draft. LT critically revised the manuscript and confirmed the final version of it to submit. All authors have reviewed and approved the final manuscript.

## Conflict of Interest

The authors declare that the research was conducted in the absence of any commercial or financial relationships that could be construed as a potential conflict of interest.

## Publisher’s Note

All claims expressed in this article are solely those of the authors and do not necessarily represent those of their affiliated organizations, or those of the publisher, the editors and the reviewers. Any product that may be evaluated in this article, or claim that may be made by its manufacturer, is not guaranteed or endorsed by the publisher.

## Footnotes

1
http://www.thomaskoenig.ch/index.php/software/

## Abbreviations

EEG: electroencephalography
FE-SCH, drug-naïve: first-episode schizophrenia
HCs: healthy controls
PANSS: Positive and Negative Syndrome Scale
VEOG: vertical electrooculogram
ANOVA: analysis of variance
rm-ANOVA: repeated measures analysis of variance.
